# Prophylactic rivaroxaban in the early post-discharge period reduces the rates of hospitalization for atrial fibrillation and incidence of sudden cardiac death during long-term follow-up in hospitalized COVID-19 survivors

**DOI:** 10.3389/fphar.2023.1093396

**Published:** 2023-05-30

**Authors:** Lukas Fiedler, Lukas J. Motloch, Anna-Maria Dieplinger, Peter Jirak, Paruir Davtyan, Diana Gareeva, Elena Badykova, Marat Badykov, Irina Lakman, Aleksandr Agapitov, Liana Sadikova, Valentin Pavlov, Fabian Föttinger, Moritz Mirna, Kristen Kopp, Uta C. Hoppe, Rudin Pistulli, Benzhi Cai, Baofeng Yang, Naufal Zagidullin

**Affiliations:** ^1^ University Department of Internal Medicine II, Cardiology and Internal Intensive Care Medicine, Paracelsus Medical University, Salzburg, Austria; ^2^ Department of Internal Medicine, Nephrology and Intensive Care Medicine, Hospital Wiener Neustadt, Wiener Neustadt, Austria; ^3^ Nursing Science Program, Institute for Nursing Science and Practice, Paracelsus Medical University, Salzburg, Austria; ^4^ Medical Faculty, Johannes Kepler University Linz, Linz, Austria; ^5^ Department of Internal Diseases, Bashkir State Medical University, Ufa, Russia; ^6^ Scientific Laboratory for the Socio-Economic Region Problems Investigation, Ufa University of Science and Technology, Ufa, Russia; ^7^ Department of Urology, Bashkir State Medical University, Ufa, Russia; ^8^ Department of Cardiology I, Coronary and Peripheral Vascular Disease, Heart Failure, University Hospital Muenster, Muenster, Germany; ^9^ Department of Pharmacology (The Key Laboratory of Cardiovascular Medicine Research, Ministry of Education), College of Pharmacy, Harbin Medical University, Harbin, China; ^10^ Department of Biomedical Engineering, Ufa University of Science and Technology, Ufa, Russia

**Keywords:** atrial fibrillation, rivaroxaban, COVID-19, post-acute COVID-19 sequelae, long COVID-19, SCD, NOAC

## Abstract

**Introduction:** While acute Coronavirus disease 2019 (COVID-19) affects the cardiovascular (CV) system according to recent data, an increased CV risk has been reported also during long-term follow-up (FU). In addition to other CV pathologies in COVID-19 survivors, an enhanced risk for arrhythmic events and sudden cardiac death (SCD) has been observed. While recommendations on post-discharge thromboprophylaxis are conflicting in this population, prophylactic short-term rivaroxaban therapy after hospital discharge showed promising results. However, the impact of this regimen on the incidence of cardiac arrhythmias has not been evaluated to date.

**Methods:** To investigate the efficacy of this therapy, we conducted a single center, retrospective analysis of 1804 consecutive, hospitalized COVID-19 survivors between April and December 2020. Patients received either a 30-day post-discharge thromboprophylaxis treatment regimen using rivaroxaban 10 mg every day (QD) (Rivaroxaban group (Riva); *n* = 996) or no thromboprophylaxis (Control group (Ctrl); *n* = 808). Hospitalization for new atrial fibrillation (AF), new higher-degree Atrioventricular-block (AVB) as well as incidence of SCD were investigated in 12-month FU [FU: 347 (310/449) days].

**Results:** No differences in baseline characteristics (Ctrl vs Riva: age: 59.0 (48.9/66.8) vs 57 (46.5/64.9) years, *p* = *n*.s.; male: 41.5% vs 43.7%, *p* = *n*.s.) and in the history of relevant CV-disease were observed between the two groups. While hospitalizations for AVB were not reported in either group, relevant rates of hospitalizations for new AF (0.99%, *n* = 8/808) as well as a high rate of SCD events (2.35%, *n* = 19/808) were seen in the Ctrl. These cardiac events were attenuated by early post-discharge prophylactic rivaroxaban therapy (AF: *n* = 2/996, 0.20%, *p* = 0.026 and SCD: n = 3/996, 0.30%, *p* < 0.001) which was also observed after applying a logistic regression model for propensity score matching (AF: *χ* ^2^-statistics = 6.45, *p* = 0.013 and SCD: *χ* ^2^-statistics = 9.33, *p* = 0.002). Of note, no major bleeding complications were observed in either group.

**Conclusion:** Atrial arrhythmic and SCD events are present during the first 12 months after hospitalization for COVID-19. Extended prophylactic Rivaroxaban therapy after hospital discharge could reduce new onset of AF and SCD in hospitalized COVID-19 survivors.

## Graphical Abstract

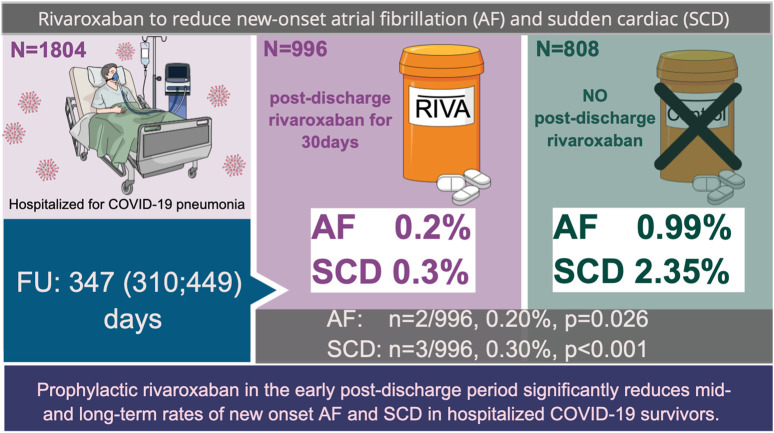

## 1 Introduction

In late 2019, the novel severe acute respiratory syndrome coronavirus-2 (SARS-CoV-2) emerged. COVID-19 disease spread rapidly worldwide and was classified by the World Health Organization (WHO) in March 2020 as a global pandemic. SARS-CoV-2 affects primarily the respiratory tract by causing bilateral pneumonia and acute respiratory syndrome associated with increased mortality rates (Connors and Levy, 2020). CV events due to coagulopathy and COVID-19-induced vascular inflammation are common and often fatal complications. The pathogenic mechanism of SARS-CoV-2 infection has been established with its spike glycoprotein binding to the angiotensin-converting enzyme 2 (ACE-2) receptor of host cells (Lu et al., 2020). For this reason, cells presenting a high expression of the ACE-2 receptor, such as myocardial cells, endothelial and artery smooth muscle cells and type II alveolar cells, amongst others, are potentially affected by SARS-CoV-2 (Zou et al., 2020). Prominent expression of ACE-2 on CV cells could explain the impact of SARS-CoV-2 infection on the CV system even in patients with low risk of CV disease (Xie et al., 2022). Cardiac injury, with consequent arrhythmogenic potential and thromboembolic complications are COVID-19 disease-specific acute manifestations in the CV system (Jirak et al., 2021a; Jirak et al., 2021b; Zagidullin et al., 2021; Jirak et al., 2022). This might be manifested in profound inflammatory response, together with endothelial inflammation and dysfunction, so-called thromboinflammation or immunothrombosis (Engelmann and Massberg, 2013; Delabranche et al., 2017; Jackson et al., 2019). Cytokines induce inflammatory effects leading to activation of vascular endothelial cells and endothelial injury which results in increasingly prothrombotic properties (Iba and Levy, 2018; Iba and Levy, 2019). Studies of survivors infected with other corona viruses have revealed palpitations caused by sinus tachycardia and arrhythmias like AF (Lau et al., 2005; Yu et al., 2006). In patients hospitalized for COVID-19, cardiac arrhythmias such as AF, significant bradyarrhythmia and non-sustained ventricular tachycardia (NSVT) were observed with an incidence between ∼6–17% (Bhatla et al., 2020; Wang et al., 2020; Wollborn et al., 2022). Arrhythmias are more likely to be seen in patients admitted to the intensive care unit (ICU) with AF at particularly high incidence (∼44%) (Bhatla et al., 2020; Colon et al., 2020).

While cardiac arrhythmias during acute COVID-19 disease are an established finding, further evidence also suggests dysrhythmias as part of the post-acute sequelae of COVID-19. Xie et al. showed a composite of dysrhythmia outcomes with HR of 1.69 among COVID-19 patients compared to non-COVID-19 patients, and an excess burden of 19.86 (18.31, 21.46) per 1000 COVID-19 survivors. Of note, the authors observed an increased risk [HR = 1.71 (1.64, 1.79) and burden of AF 10.74 (9.61, 11.91)] (Xie et al., 2022).

In long-term sequelae of COVID-19, multiple organ systems including the CV system stay affected and symptoms persist over a longer period indicating an urgent need for reliable therapy options (Michelen et al., 2021). In the Russian Federation during the pandemic, direct oral anticoagulants (DOAC) were prescribed in several medical centers for 30 days after hospitalization for COVID-19. A prophylactic DOAC strategy was adopted in a nationwide class C guideline recommendation in 2020 (Russian Ministry of Healthcare, 2020). This approach is supported by the results of a large United States registry in post-discharge COVID-19 patients, showing 46% reduction in thromboembolic events with prophylactic anticoagulation over 90 days FU (Giannis et al., 2021) as well as by the results of the recently published MICHELLE trial where prophylactic rivaroxaban for 35 days in high-risk COVID patients with high D-dimers reduced a composite of thrombotic events and death by 67% (Ramacciotti et al., 2022).

Nevertheless, to the best of our knowledge, the effects of prolonged anticoagulation on arrythmias and SCD in hospitalized COVID-19 survivors have not been evaluated to date. Moreover, longer FU data on arrhythmogenic outcome in survivors after COVID-19 hospitalizations are still rare in the literature. To investigate this issue, we assessed the incidence post-hospitalization for newly diagnosed AF and higher degree AVB as well as rates of SCD during median FU of 347 days in 1804 hospitalized COVID-19 survivors stratified by receipt of prophylactic rivaroxaban for 30 days post-discharge. We hypothesized, that the applied therapy regimen would affect post COVID-19 sequelae and influence incidence of arrhythmias requiring hospitalization and thus potentially cardiac death rates.

## 2 Materials and methods

### 2.1 Study design

The present study was performed in accordance with standards of good clinical practice and the principles of the Declaration of Helsinki. The study protocol was approved by the Local Ethical Committee (N5, 2020). Prior to inclusion, all participants signed an informed consent.

In this single-center, retrospective study at a tertiary medical university center in the Russian Federation, we screened 2,294 consecutive COVID-19 survivors who were hospitalized between April 2020 and December 2020 for moderate COVID-19 pneumonia, defined according to current World Health Organization (WHO) recommendations (WHO, 2020)[26]. COVID-19 survivors were defined as patients who survived hospitalization for moderate COVID-19 pneumonia and were discharged alive from the hospital.

All included patients were 18 years or older and had presented with moderate COVID-19-related pneumonia requiring hospitalization. Exclusion criteria were defined as: history of AF or other pathologies which required therapeutic anticoagulation using Vitamin K antagonists, heparinoids or DOAC therapy before enrollment, including history of relevant thrombotic disorders requiring anticoagulation therapy. Although at the beginning of the pandemic dipyridamole was also prescribed in our center as a standard antithrombotic regime in hospitalized COVID-19 patients after hospital discharge, patients receiving this treatment were excluded from further analyses. In the present trial, we decided to investigate the effect of Rivaroxaban only. Thus, patients treated with other prophylactic DOAC (edoxaban, apixaban and dabigatran) or heparins regimes upon discharge were also excluded. Concomitant treatment with antithrombotics including acetylsalicylic acid (ASA), clopidogrel, prasugrel or ticagrelor was not an exclusion criterion. To account for disease severity and associated arrhythmic burden, patients with severe COVID-19 pneumonia (WHO, 2020) including those with requirement for mechanical ventilation support, defined as the need for endotracheal intubation and/or the need for non-invasive ventilation including mechanical ventilation involving end-expiratory and inspiratory positive air pressure support pressure via a tightly fitted face mask/helmet or the usage of High Flow Nasal Oxygen Canula were excluded from further analyses.

Choice of the anticoagulation therapy for 30-day post hospital discharge (none vs rivaroxaban 10 mg) was based on the decision of the attending physician and implemented according to hospital-specific standard of care procedures with respect to the patient’s will. All COVID-19 related in-hospital treatments provided within the period of 1 month after hospital discharge (including prophylactic anticoagulation) were financially covered by state insurance. Furthermore, to handle difficult access to drugs during the pandemic situation, COVID-19 related medication (including prophylactic anticoagulation) was partly delivered to the patients by volunteers or provided in the hospital at discharge.

Patients’ hospital data including demographics, medical history, laboratory examinations, comorbidities, complications, specific treatment measures, and outcomes were captured. During patients’ FU, the incidence of newly diagnosed AF requiring hospitalization and higher degree AVB requiring hospitalization as well as the incidence of sudden cardiac death were evaluated. Sudden cardiac death was defined as in-hospital death during hospitalization requiring resuscitation due to cardiac causes, or out of hospital death meeting the criteria of sudden cardiac death (Zeppenfeld et al., 2022). Diagnosis was based on screened death certificates. Higher degree AVB was defined as AVB type Mobitz IIb and III or type Mobitz IIa requiring pacemaker therapy according to current ESC guidelines (Glikson et al., 2021).

Furthermore, patients were evaluated for major bleeding, defined according to International Society on Thrombosis and Haemostasis (ISTH) criteria (Kaatz et al., 2015). Study endpoints were investigated until 1 October 2021 using the remote data capture system “ProMed” (Program for Medical Cases Monitoring). The program enables distant online monitoring of all hospitalization discharge notes from all regional hospital institutions as well as all death certificates. At the time of FU data collection (after 1 October 2021), all patients were contacted by phone. A standardized telephone interview was conducted to verify the applied anticoagulation therapy (none vs rivaroxaban) and the patient’s drug adherence. If a patient was deceased by the time of scheduled contact, a standardized telephone interview was performed with a close relative to confirm drug adherence in patients who did not survive the FU. Patients with requirement for therapeutic anticoagulation including the application of any DOAC in therapeutic dose, Vitamin K antagonists or heparinoids were excluded from further analyses. Patients were also excluded from further analyses if collection of sufficient information about the therapy regime was not possible. These patients were counted as lost to FU, which occurred in 16 cases.

Based on the described inclusion and exclusion criteria, 1804 COVID-19 patients were included in the present study. Ctrl (*n* = 808) was defined based on the described inclusion and exclusion criteria as patients who were not treated during FU with any prophylactic or therapeutic anticoagulation regime including the application of any DOAC, Vitamin K antagonists or any heparins independently of the application of any antithrombotic therapy regime including ASA, clopidogrel, prasugrel or ticagrelor. The Riva group (*n* = 996) was defined based on the inclusion and exclusion criteria as patients who were treated with prophylactic dose of Rivaroxaban for 30-day post hospital discharge independently of the application of any antithrombotic therapy regime including ASA, clopidogrel, prasugrel or ticagrelor.

### 2.2 Statistical analyses

Primarily all data was tested for normal distribution using the Shapiro–Wilk test. If normally distributed, continuous variables were presented as mean values M) and standard deviations (SD), or if non-normally distributed, data were expressed as medians and interquartile range Q1–Q3. The Mann-Whitney test was used to assess differences in continuous variables between groups while the Chi-square test was used to analyze categorical variables. The test was corrected for likelihood if the rate of occurrence did not exceed 2%. Otherwise, the Yate’s correction was applied.

To reduce selection bias caused by the lack of randomization, the statistical method of pseudo-randomization was applied using propensity score matching. Consequently, in the next step, variables showing statistically significant differences as well as the variable male gender were selected as confounder candidates and included in the initial pseudo-randomization procedure. Differences were considered statistically significant if *p* < 0.050.

Propensity score matching was carried out by a logit model using confounder candidates as regressors. Distribution of candidates into the Ctrl or the Riva group was chosen as the target variable. The propensity score index of distribution into each group was calculated as a result of the logit model evaluation. The obtained results of the logit model were further confirmed by the method of correctness of choice of variables as confounders. In this case, *p* < 0.100 was regarded as statistically significant. The method « Nearest» was applied by dividing the entire sample into strata Ctrl or the Riva group to control the balances of propensity score distribution. This procedure enabled equitation of the volumes of both investigated cohorts after balancing. Finally, a log-rank test was applied to assess statistical differences in Ctrl vs Riva groups for the investigated study endpoints. Furthermore, to confirm our results, Kaplan-Meier multiplier scores were calculated for the investigated study endpoints (hospitalization for new-onset AF and SCD) for both groups (Ctrl vs. Riva) and corrected for the established cofounders.

A *p*-value <0.050 was considered as statistically significant. All statistical analyses were assessed using R software (version 3.6.3, R Foundation for Statistical Computing, Vienna, Austria, https://www.r-project.org).

## 3 Results

In total, 1,804 patients (100% Caucasian) were included in the final statistical analysis. While a prophylactic oral anticoagulation therapy using rivaroxaban 10 mg QD for 30-day after hospitalization was used in 996 patients, 808 patients received no anticoagulation therapy and served as Ctrl. Baseline characteristics and laboratory values at the time of enrollment are depicted in Table 1. During the in-hospital period, all patients were treated at minimum with prophylactic anticoagulation therapy using a heparinoid while the majority also received therapeutic anticoagulation (73.5%). Patients in the Riva group were older and showed a higher prevalence of arterial hypertension (AH) which resulted in higher rates of aldosterone-antagonist usage at hospital discharge, while patients in Ctrl showed a higher rate of acetylsalicylic acid therapy usage. During hospitalization, higher CRP levels were observed in Riva patients indicating a higher inflammatory burden in this population. Furthermore, a higher implementation of COVID-19-specific treatments including corticosteroids, IL6-antagonist and therapeutic anticoagulation therapy was observed in the Riva group.

**TABLE 1:** Baseline characteristics of the study population.

	Riva + control (n = 1804)		Riva (n = 996)		Control (n = 808)		*p*-value	Correction
	%	n	%	n	%	n		-
Follow-up								-
BMI, kg/m2		27.9 (25.7; 31.8)		27.7 (25.2; 31.6)	-	28.0 (25.8; 32.0)	0.342	-
Hospital stay, days		11 (9; 13)		11 (8; 13)	-	11 (9; 13)	0.778	-
Age		58.3 (47.5; 66.5)		59.0 (48.9; 66.8)	-	57 (46.5; 64.9)	0.0237*	-
Sex (male/female)	42.6/57.4	770/1034	43.7/56.3	435/561	41.5/58.5	335/473	0.345	-
Lung damage, %		40 (28; 52)		40 (28; 52)	-	36 (25; 52)	0.2924	-
Arterial hypertension	37	674	39.5	393	34.7	281	0.041*	-
Diabetes mellitus	11	203	11.8	118	10.5	85	0.954	-
Chronic kidney disease	3.1	56	3.1	31	3.09	25	0.983	-
Coronary heart disease	7.3	13	7.4	74	7.1	58	0.834	-
Heart failure	6.3	115	6.3	63	6.4	52	0.924	-
COPD	3.3	59	3.1	31	3.4	28	0.676	-
Laboratory data								-
Hb (mg/dL)		13.3 (12.3; 14.1)		13.35 (12.4; 14.2)	-	13.2 (12.3; 14.1)	0.0720	-
GFR (MDRD) (mL/min/1,73 m2)		67.6 (57.5; 79.1)		68 (57.6; 79.2)	-	66.8 (57.2; 79)	0.4607	-
CRP (mg/L)		24 (0; 58.3)		27.3 (6.0; 59.1)	-	22 (0; 57)	0.0301*	-
Sodium (mmol/L)		143 (141; 144)		143 (141; 144)	-	143 (141; 144)	0.3163	-
Potassium (mmol/L)		4.2 (3.9; 4.5)		4.2 (3.9; 4.5)	-	4.2 (3.9; 4.4)	0.6268	-
In-hospital therapy								-
Corticosteroids	86.7	1567	91.2	909	81.4	658	<0.001***	-
Therapeutic anticoagulation	73.5	1326	82.8	825	62	501	<0.001***	-
JAK-inhibitors	10.14	183	11.1	111	8.9	72	0.103	-
IL6-antagonist	74.2	1339	77.4	771	70.2	568	<0.001***	-
Hydroxychloroquine	1.39	25	1.2	12	1.6	13	0.506	-
Azythromycin	1.39	25	1.2	12	1.6	13	0.506	-
Discharge therapy								-
Statins	7.7	139	8.3	83	6.9	56	0.267	-
Betablockers	12.2	221	12.2	122	12.2	99	0.999	-
ACE/ARB	21.6	389	22.1	221	20.7	168	0.474	-
Verapamil/Diltiazem	0.6	11	0.7	7	0.49	4	0.572	Likelihood
Diuretics	4.5	81	4.7	47	4.2	34	0.603	-
Sotalol	0.2	3	0.3	3	0	0	0.327	Yate’s correction
ASA	4.0	72	2.4	24	5.9	48	<0.001***	-
Clopidogrel	0.4	8	0.3	3	0.6	5	0.314	Likelihood
MRA	27.1	490	34.2	341	18.3	148	<0.001***	-
Digoxine/Digitoxine	0.06	1	0	0	0.1	1	0.917	Yate’s correction
Ivabradine	0.39	7	0.3	3	0.5	4	0.512	Likelihood

Baseline characteristics of enrolled patients. ACE, angiotensin-converting enzyme inhibitors; ARB, angiotensin receptor blockers; ASA , acetylsalicylic acid; BMI, body mass index; COPD, chronic obstructive pulmonary disease, CRP = c-reactive protein, GFR, glomerulation filtration rate, IL6 = Interleukine-6, JAK = janus kinase; MRA, mineralocorticoid receptor antagonist; *, **, *** - significance in *p* < 0.05, *p* < 0.01, *p* < 0.001.

Mean FU in the total cohort was 347 (310; 449) days. During entire FU period, no major bleeding events occurred in either group. Furthermore, we did not observe any hospitalizations for higher degree AVB.

However, in comparison to Riva, a higher rate of hospitalization for new-onset AF was observed in the Crtl group starting at 6 months post-discharge, which remained significantly higher during the total FU period (Table 2): rates of hospitalization for new-onset AF at the end of FU Riva: 0.20% (*n* = 2) vs Ctrl: 0.99% (*n* = 8), *p* = 0.022]. Furthermore, in the Ctrl group, a high and steady rate of SCD (2.35%) during total FU was observed. Of note, these events were decreased in Riva, when matched to Ctrl. These observed differences remained consistent during the total FU period (Table 3): rates of SCD at 30 days [Riva: 0.00% (*n* = 0) vs Ctrl: 0.62% (*n* = 5), *p* = 0.042], rates of SCD at 6 months [Riva: 0.10% (*n* = 1) vs Ctrl: 1.36% (*n* = 11), *p* < 0.001] and rates of SCD at the end of FU [Riva: 0.30% (*n* = 3) vs Ctrl: 2.35% (*n* = 19), *p* < 0.001].

**TABLE 2:** New-onset atrial fibrillation during follow-up after hospitalization due to COVID-19.

FU	Riva + Control (n = 1804)		Riva (n = 996)		Control (n = 808)		Correction	*p*-value
	%	n	%	n	%	n		
30-day	0.06	1	0	0	0.12	1	Yate’s correction	0.917
6-month	0.5	9	0.2	2	0.87	7	Likelihood	0.043^a^
AF during total Follow-up 347 (310; 449) days	0.55	10	0.2	2	0.99	8		**0.022^a^**

AF, hospitalization for new-onset atrial fibrillation; COVID-19, Coronavirus disease 2019; Riva = rivaroxaban.

^a^ , **, *** - significance in *p* < 0.05, *p* < 0.01, *p* < 0.001.

**TABLE 3:** Sudden cardiac death during follow-up after hospitalization due to COVID-19.

	Riva + control (n = 1804)		Riva (n = 996)		Control (n = 808)		Correction	*p*-value
	%	n	%	N	%	n		
30-day SCD	0.28	5	0	0	0.62	5	Yate’s correction	0.042^a^
6-month SCD	0.67	12	0.1	1	1.36	11	Likelihood	<0.001***
SCD during total Follow-up 347 (310; 449) days	1.16	21	0.3	3	2.35	19		<0.001***

SCD, sudden cardiac death; COVID-19, Coronavirus disease 2019; Riva = rivaroxaban.

^a^ , **, *** - significance in *p* < 0.05, *p* < 0.01, *p* < 0.001.

We further decided to confirm our observations by applying a propensity score matching using a logit model with confounder candidates as regressors, as described above. Consequently, variables showing significant differences between groups (*p* < 0.050) as well as the variable male gender were selected initially as independent factors in the logistic regression model for propensity score matching. These included: age, AH, rates of aldosterone-antagonist therapy after discharge, rates of acetylsalicylic acid therapy after discharge, rates of in-hospital corticosteroid therapy, rates of in-hospital IL6-antagonist therapy and rates of in-hospital therapeutic anticoagulation therapy as well as CRP-levels at hospital admission. In the next step, the obtained results of the logit model were confirmed by the method of correctness of choice of variables as confounders (Supplementary Table S1). By applying this correction, the variables AH, rates of in-hospital corticosteroid therapy, rates of in-hospital therapeutic anticoagulation therapy, rates of aldosterone-antagonist therapy after discharge and rates of acetylsalicylic acid therapy after discharge remained as relevant variables for sample balancing as they displayed statistically significant values (*p* < 0.100; Supplementary Table S1).

In the next step, a pseudo-randomization based on the obtained results was applied, which resulted in reduction of the sample size of the Riva group (*n* = 808 while 168 observations were removed from the Riva group). Furthermore, a log-rank test model to determine differences in the interval before the onset of hospitalization for new-onset of AF and new onset of SCD during total FU after hospital discharge was calculated for Riva versus Ctrl. Of note, the results revealed significant differences for both evaluated study endpoints (Table 4): hospitalization for new-onset AF (χ2-statistics = 6.45, *p* = 0.013) and SCD (χ2-statistics = 9.33, *p* = 0.002).

**TABLE 4:** Results of study endpoints comparison during total follow-up after applying logistic regression model for propensity score matching.

	χ^2^-statistics	Р-level
Hospitalization for new-onset AF	6.45	0.013*
Sudden cardiac death	9.33	0.002**

*, **—significance in *p* < 0.05, *p* < 0.01.

**FIGURE 1:**
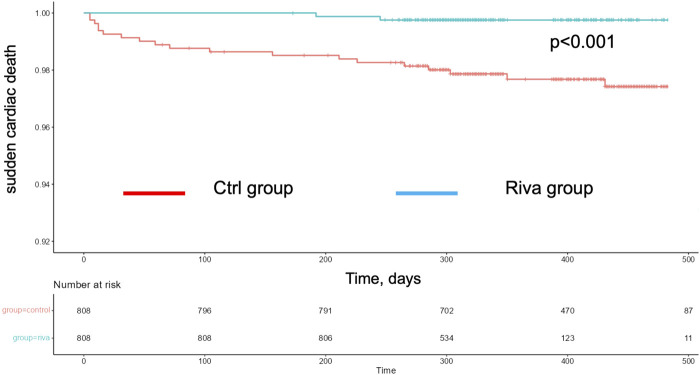
Corrected Kaplan-Mayer curves for hospitalization for new-onset AF curves in rivaroxaban (green) and control (red) group.

**FIGURE 2:**
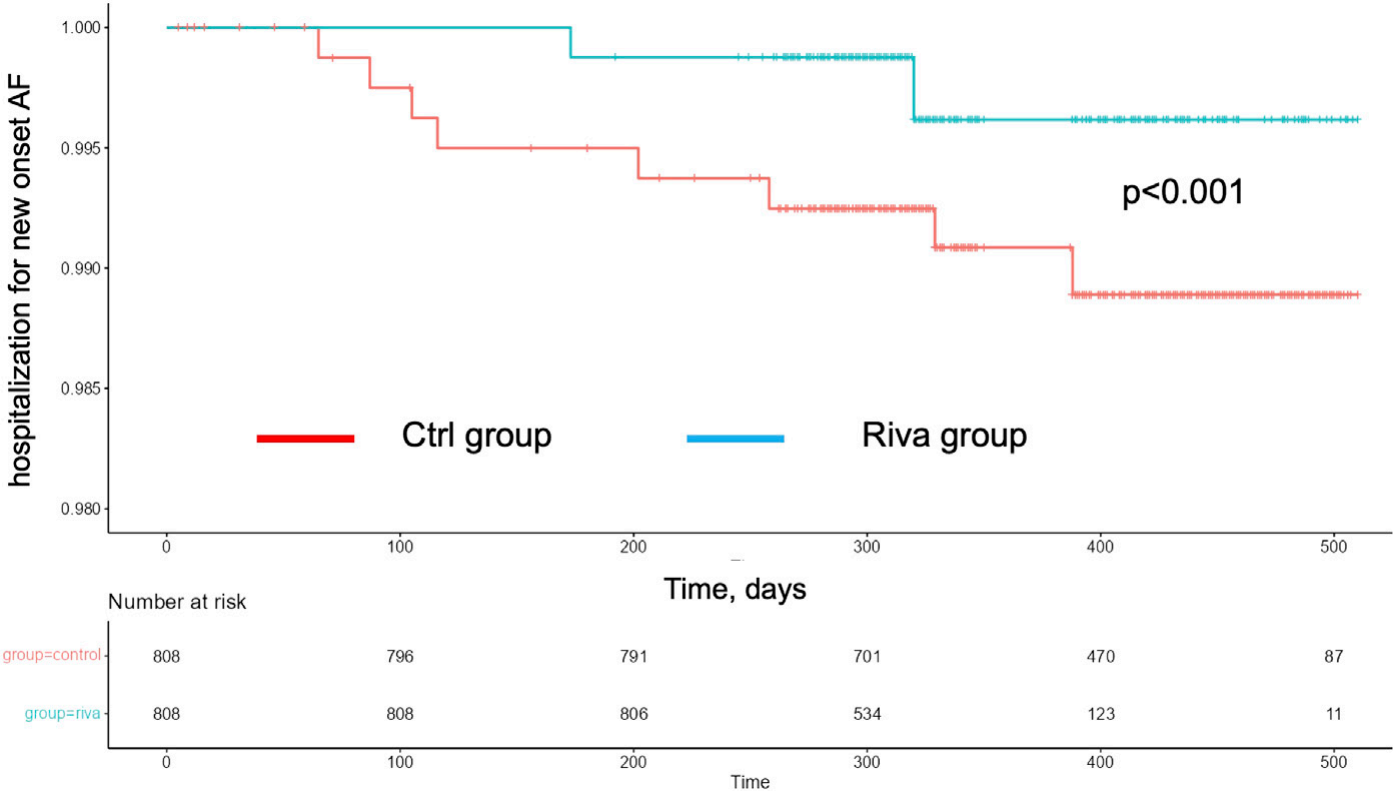
Corrected Kaplan-Mayer curves for sudden cardiac death in rivaroxaban (green) and control (red) group.

To confirm our results, rates of hospitalization for new-onset AF and SCD rates were further evaluated by calculation of Kaplan-Meier multiplier scores, which included the above mentioned cofounders. Of note, when Ctrl was matched to Riva, corrected Kaplan-Meier analyses during the total FU revealed highly significant differences for both endpoints: rates of hospitalization for new-onset of AF (Figure 1, *p* < 0.001) as well as rates of SCD (Figure 2, *p* < 0.001).

## 4 Discussion

The COVID-19 pandemic led to a burgeoning population of patients recovering from SARS-CoV-2 infection and is still affecting healthcare systems worldwide. The impact of acute COVID-19 on the human organism is progressively understood but growing observational data suggest that patients present with a wide range of persisting symptoms after recovery from acute illness (Goërtz et al., 2020; Xiong et al., 2021). Despite consistent research with advances in therapy and vaccination measures, COVID-19 and COVID-19-sequelae is likely to remain a considerable problem for healthcare systems worldwide. Treatment and prevention of long-term sequelae of COVID-19 still pose a major challenge. Thus, better understanding of pathophysiology and treatment in the context of long-term COVID-19 is of utmost importance.

SARS-CoV-2 infection mainly affects the respiratory system but additionally causes cardiac injury in over 20% of cases. An increased risk of AF with a HR of 1.57 (95% CI 1.23, 2.00) is reported for COVID-19 patients (Wollborn et al., 2022). A metanalysis showed 14.24 (95% CI: 8.67–23.38) higher odds for mortality in acute COVID-19 patients with acute cardiac injury compared to COVID-19 patients without acute cardiac injury (Toloui et al., 2021). During hospitalization, patients with cardiac injury more frequently presented with malignant arrhythmias (11.5% vs. 5.2%) including ventricular tachycardia and ventricular fibrillation (Guo et al., 2020). For patients with long-COVID sequelae, an increased risk of AF [HR = 1.71 (1.64, 1.79)] and an increased risk for ventricular arrhythmias [HR = 1.84 (1.72, 1.98)] have been reported (Xie et al., 2022).

CV complications of severe inflammatory states have been held responsible for new onset of AF and ventricular tachycardia (Ambrus et al., 2015; Klein Klouwenberg et al., 2017; Bhatla et al., 2020) Development of palpitations and/or diverse cardiac arrhythmias were shown to be frequent in acute and post COVID-19 infection, accounting for 9%–14% of CV symptoms in several observational studies (Babapoor-Farrokhran et al., 2020; Bhatla et al., 2020; Kochav et al., 2020; Carvalho-Schneider et al., 2021; Huang et al., 2021; Zhou et al., 2021). Besides AF, the incidence of ventricular arrhythmias in patients hospitalized for COVID-19 is also significantly increased (Guo et al., 2020). Consequently, a concomitant increase in cardiac arrests associated with COVID-19 has been observed (Bhatla et al., 2020) Therefore, further investigations of preventive strategies in this patient population are warranted.

To further analyze this issue, we evaluated dysrhythmias in patients post-discharge after hospitalization for COVID-19. In this cohort, we performed a retrospective long-term FU [FU: 347 (310; 449) days] evaluating rehospitalization with new onset AF, occurrence of higher degree AVB as well as incidence of SCD in 1,804 patients hospitalized for COVID-19. With respect to higher AVB, no differences were observed between the groups. However, while no major bleeding events were reported in either group, we were able to demonstrate, that during longer FU, administration of prophylactic rivaroxaban in the early post-discharge period might reduce hospitalization rates for new onset of AF and SCD events. In our study, these observations were reinforced by application of a propensity score matching model. Thus, to the best of our knowledge, for the first time, our results could hint at antiarrhythmic effects of the investigated prophylactic therapy regime upon post COVID-19 sequelae.

While potential antiarrhythmic implications of an anticoagulation therapy might seem hard to explain, our observation could be partially elucidated by previous findings. Recent data hints at pleiotropic antiarrhythmic actions of this substance class. Beyond others, FXa inhibition includes effects on electrical and structural remodeling in the heart as well as on inflammatory components of the vulnerable substrate, which are not related to hemostasis (Fender et al., 2019). There is growing evidence in the bidirectional relationship between coagulation and AF, where AF can cause a hyper-coagulant state, and an altered hemostatic balance can also contribute to the development and progression of AF. In a recent metanalysis, the authors found a significant association between fibrinogen plasminogen activator inhibitor 1 (PAI-1), and D-dimer and AF incidence (Chang et al., 2013). Rivaroxaban directly abbreviates left atrial action potential duration and increases both L-type Ca2^+^-current and ultra-rapid delayed rectifier K^+^ current in isolated left atrial cardiomyocytes, without affecting transient outward K^+^ current (Chang et al., 2013). Notably, a direct inhibitory effect of Rivaroxaban on FXa/PAR2-mediated fibro-inflammatory processes has been shown in human atrial tissue slices (Bukowska et al., 2013), with Rivaroxaban halting the feed-forward upregulation of PAR2 much as is seen in other cell types (Jobi et al., 2011). Importantly, while direct thrombin inhibition prevented left atrial remodeling and AF (Jumeau et al., 2016), in further trials, Rivaroxaban was able to reduce spontaneous electrical activities in pulmonary veins indicating an additional antiarrhythmic effect of this substance (Chang et al., 2018).

Regarding arrhythmia pathogenesis in COVID-19, Musikanow et al. showed that in patients hospitalized with severe COVID-19 presenting without a history of atrial arrhythmias, new onset of AF occurred in 4% of the patient population and is therefore comparable to other viral diseases (Musikantow et al., 2021). It is well known that thrombin-induced secretion of proinflammatory cytokines and growth factors play a key role in coagulation-induced inflammation (Kenan et al., 2012). In a healthy individual, thrombin combined with thrombomoduline activates protein C at the endothelium. Activated protein C (APC) provides an anticoagulation effect on the one hand but also substantial anti-inflammatory effects (Feistritzer et al., 2005; Esmon, 2012). Therefore, one might speculate that DOAC therapy could possibly lessen the reciprocal interaction between inflammation and thrombosis in COVID-19 (Bikdeli et al., 2020; Paar et al., 2021). Indeed, Martins et al. could demonstrate that in patients with AF, the use of Rivaroxaban was associated with a decrease of inflammatory cytokines in comparison to warfarin (Martins et al., 2020). This might raise the hypothesis that the anti-inflammatory effect of Rivaroxaban is potentially based on a direct factor Xa (FXa) inhibition (Steinberg, 2005). Of note, in previous trials, patients with AF and SCD presented with elevated plasma-levels of inflammatory cytokine (Hadi et al., 2010; Li et al., 2010; Landfors et al., 2022). Those cytokines are the basis of pathogenesis, maintenance, and recurrence of arrhythmias (Hu et al., 2015). FXa mediates inflammatory signaling in atrial tissue, possibly by the activation of protease-activated receptors (Bukowska et al., 2013). Reduction of pentraxin-related protein (PTX-3) functions as a reliable marker to determine the anti-inflammatory effect of FXa inhibitors, which seem to be another axis of an anti-inflammatory effect (Hu et al., 2015). As FXa inhibitors have been demonstrated to have anti-inflammatory effects in addition to their anticoagulant effects (Terry et al., 2016; Katoh et al., 2017), it might be assumed that this effect would reduce AF and SCD. However, as inflammatory burden was not evaluated during FU in our study, these speculations should be viewed with caution.

Thus, also other explanations should be considered. New onset of atrial arrhythmias was associated with an increased mortality in the literature. (Berman et al., 2020; Mountantonakis et al., 2021; Musikantow et al., 2021). This has also been demonstrated in patients infected with SARS-CoV-2 (Zhao et al., 2022) Thus, anti-inflammatory and anticoagulatory effects of Rivaroxaban might also lead to improvement in CV outcomes with consequent reduction of CV mortality. A recent trial studied these potential implications of using Rivaroxaban 10 mg QD, as prolonged thromboprophylaxis versus control in COVID-19 patients after hospital discharge. Indeed, the primary composite outcome of thromboembolic complication, myocardial infarction, stroke, and CV death, was significantly reduced in the Riva group compared to the Ctrl after a treatment period of 35 days (Ramacciotti et al., 2022). While rivaroxaban was only given in the early post-discharge period, potential antiarrhythmic effects were observed after a longer FU period. Thus, our results could indicate that early medical intervention is crucial in reducing arrhythmic burden in hospitalized COVID-19 survivors. Our speculations might be supported by previous results. Although, acute COVID-19 persists for a limited time-period, an increased incidence of AF and SCD was reported after 1 year (Xie et al., 2022). This is consistent with trials in COVID-19 survivors reporting prolonged coagulation abnormalities (Wang et al., 2022), longer virus persistence (Sun et al., 2020) and enhanced inflammatory processes during long-term FU. Interestingly, long-term cardiovascular complications seem to be associated with the severity of acute COVID-19 disease (Xie et al., 2022) which is characterized by a high inflammatory burden with increased coagulopathy predominantly observed in the first 30 days (Demelo-Rodríguez et al., 2021). Besides their established anticoagulant activity, FXa inhibitors seem to possess anti-inflammatory and antiviral potential (Al-Horani, 2020). Consequently, one might speculate persistent long-lasting effects when prescribed for a short-term period in the early phase of COVID-19 disease which is characterized by an increased inflammatory burden. This speculation is supported by a recent report from the SARCOV-19 registry evaluating 30 days of anticoagulation in COVID-19 patients upon hospital discharge. Of note, consistent with our data, the authors were able to demonstrate a reduction of thromboembolic events during a FU of 1 year (Aidouni et al., 2023). While the described results seem only hypothesis-generating, the large ongoing double-blind, placebo-controlled randomized control ACTIV-4c trial (NCT04650087) is currently evaluating midterm cardiovascular outcomes (90 days) after a 30 days regime of prophylactic apixaban started upon hospital discharge (Ortel, 2023). Our observations are further supported by previous reports indicating higher long-term CV risk post-discharge following acute COVID-19 infection (Whiteley and Wood, 2022) as well as trials indicating persistent inflammatory processes and virus persistence in long-term FU of COVID-19 sequelae (Sun et al., 2020; Seeble et al., 2022).

There are some limitations in our study which need to be addressed. As this is an observational, retrospective, single center, real-life scenario trial, consequently with lack of randomization and blinding in the treatment arm, results need to be interpreted with caution. This might limit the reliability and validity of our data. Therefore, our findings must be considered primarily as hypothesis-generating as they are not a result of a randomized analysis. This is further emphasized by a potential increase in the probability of selection bias promoted by the strategy of anticoagulant therapy choice which in out trial was based on the physician’s decision. Another bias is loss of FU which occurred in 16 patients. Furthermore, new onset of AF was evaluated only in the framework of hospitalization events. As we did not screen for asymptomatic or ambulatory managed cardiac arrythmias, the true incidence of AF and ventricular arrhythmias was probably underestimated in our cohort. This issue was accompanied by the lack of close monitoring for medication adherence since this matter was only confirmed by a single phone interview at the end of the FU period.

Our data was collected in the early stage of the pandemic when standard medical strategies were not established with consequent usage of untested medical therapies (Zuckerman et al., 2022). Furthermore, the center-specific anticoagulation regimes began to be used as the pandemic progressed, while patients enrolled in the Ctrl were mainly treated during the very early stage of the study. SCD rates in our study seem relatively high (2.35%). One has also to consider general medical shortages during the pandemic which could possibly drive the incidence of CV pathologies. Therefore, our results must be interpreted with caution when extrapolated to an ordinary situation. Furthermore, preventive CV risk management strategies were not investigated during FU. All patients in this study suffered from COVID-19 between April to December 2020. Consequently, rapid evolution of the viral genome and COVID-19 management strategies should also be considered. However, to account for this bias, results were adjusted by propensity score matching of groups which did not significantly affect our results. In our study, bleeding events were low, which seems plausible, since comparably low results were presented in the MICHELLE study, which used a similar therapeutic regime in a comparable population of patients (Ramacciotti et al., 2022). Still, we were only able to analyze bleeding events requiring hospitalization, which represents a major limitation. Furthermore, it is of particular importance that the results of this observation alone apply to hospitalized patients with moderate COVID-19 infection. As patients with light and severe COVID-19 pneumonia were not included in our study, our results can only be applied to moderate COVID-19 cases. Furthermore, while potential anti-inflammatory and antiviral effects of Rivaroxaban were not investigated in our trial, the pathophysiology underlying our observations remains speculative and needs to be investigated in further studies. The assessment of relevant cardiac and COVID-19-related biomarkers as LDH, interleukine-6, troponins as well as NT-proBNP would promote better insight into associated pathophysiological processes. However, as our results were obtained during the first wave of the pandemic, these biomarkers were not routinely assessed in our cohort.

In summary, this study is the first to offer long-term FU (347 (310; 449) days) of prolonged Rivaroxaban therapy regime after hospitalization for COVID-19. Of note, in our study, in the absence of major bleeding events, both hospitalization rates for new-onset AF and SCD events were significantly reduced by this prophylactic treatment. This finding suggests an ongoing thromboembolic and inflammatory burden in COVID-19 survivors during the first year following the acute phase of the disease. For this reason, extended thromboprophylaxis treatment with rivaroxaban might offer additional beneficial antiarrhythmic effects in hospitalized post-COVID-19-patients post-discharge. Randomized controlled trials are necessary to evaluate the effects of these regimens in hospitalized COVID-19 survivors.

## Figures and Tables

**Table:** 

**ACE**	Angiotensin-converting enzyme
**AH**	Arterial hypertension
**AF**	Atrial fibrillation
**ARB**	Angiotensin receptor blockers
**ASA**	Acetylsalicylic acid
**AVB**	Atrioventricular-block
**CI**	Confidence interval
**Coef**	Coefficient
**CK**	Creatine kinase
**CKD**	Chronic kidney disease
**COPD**	Chronic obstructive pulmonary disease
**COVID-19**	Coronavirus disease 2019
**Ctrl**	Control group
**CRP**	C-reactive protein
**CV**	Cardiovascular
**GFR**	Glomerular filtration rate
**FU**	Follow-up
**Hb**	Hemoglobin
**ICU**	Intensive care unit
**IL-6**	Interleukin-6
**ISTH**	International Society on Thrombosis and Haemostasis
**JAK**	Janus Kinase
**M**	Mean values
**MRA**	Mineralocorticoid Receptor Antagonist
**NSVT**	Non-sustained ventricular tachycardia
**QD**	Every day
**Riva**	Rivaroxaban group
**SARS-CoV-2**	Severe acute respiratory syndrome coronavirus type 2
**SCD**	Sudden cardiac death
**SD**	Standard deviations
**WHO**	World Health Organization

## Data Availability

The raw data supporting the conclusion of this article will be made available by the authors, without undue reservation.
