# Supplementary material for: Prophylactic rivaroxaban in the early post-discharge period reduces the rates of hospitalization for atrial fibrillation and incidence of sudden cardiac death during long-term follow-up in hospitalized COVID-19 survivors
Source: Front Pharmacol. 2023 May 30;14:1093396. doi: 10.3389/fphar.2023.1093396 (PMC10266094; doi:10.3389/fphar.2023.1093396)
Supplement: Supplementary file 1 [file DataSheet1.pdf]

Supplement table 1.

Results of calculation of correctness of choice of relevant variables as confounders

Confounder	Estimate±SE	p-level
Age	-0.001 ± 0.004	0.816
Male gender	0.089 ± 0.102	0.382
Arterial hypertension	0.218 ± 0.113	0.054*
CRP-levels at admission	0.001 ± 0.002	0.499
In hospital Corticosteroid therapy	0.365 ± 0.162	0.024**
In hospital therapeutic anticoagulation therapy	0.825 ± 0.127	<0.001***
In hospital anti-IL-6 in hospital therapy	-0.050 ± 0.120	0.675
ASA therapy after discharge	-1.094 ± 0.269	<0.001***
Aldosteron Antagonist therapy after discharge	0.554 ± 0.125	<0.001***

ASA= acetylsalicylic acid, CRP=C-reactive protein, IL-6=interleukin-6; *, **, *** - significant difference from at p<0.100, p<0.050, p<0.001.

Supplemental table 2

Available levels of COVID-19 disease relevant biochemical biomarkers in Control vs. Rivaroxaban group

	Median (Q1, Q3)		p-level
	Control	Riva	
n	188	405	
IL-6, µg /mL	5.40 (1.39, 17.85)	6.18 (0.89, 24.93)	0.848
n	635	735	
LDH, U/l	358 (286.5, 444)	368.5 (301.5, 458.5)	0.021*
n	27	65	
sST2, ng/mL	45.02 (31.94, 65.41)	53.28 (35.12, 72.47)	0.440
n	27	65	
Troponin T, ng/mL	0.03 (0.01, 0.055)	0.02 (0, 0.07)	0.6770

Levels of COVID-19 disease relevant biochemical biomarkers at hospital admission; IL-6=interleukone-6, LDH= lactate dehydrogenase, sST-2= soluble ST2; data are expressed as medians and interquartile range Q1–Q3. The Man-Whitney test was used to assess differences between groups; *, **, *** - significance in p<0.05, p<0.01, p<0.001.
